# Vasoactive Management of Pulmonary Hypertension and Ventricular Dysfunction in Neonates Following Complicated Monochorionic Twin Pregnancies: A Single-Center Experience

**DOI:** 10.3390/children11050548

**Published:** 2024-05-03

**Authors:** Lukas Schroeder, Leon Soltesz, Judith Leyens, Brigitte Strizek, Christoph Berg, Andreas Mueller, Florian Kipfmueller

**Affiliations:** 1 Department of Neonatology and Pediatric Intensive Care Medicine, University Children’s Hospital Bonn, Venusberg-Campus 1, 53127 Bonn, Germanyflorian.kipfmueller@ukbonn.de (F.K.); 2 Department of Obstetrics and Prenatal Medicine, University Hospital Bonn, 53127 Bonn, Germany; 3 Division of Prenatal Medicine and Gynecologic Sonography, Department of Obstetrics and Gynecology, University of Cologne, 50931 Cologne, Germany

**Keywords:** monochorionic, twin pregnancies, pulmonary hypertension, ventricular dysfunction, vasoactive management

## Abstract

Objectives: Twins resulting from a complicated monochorionic (MC) twin pregnancy are at risk for postnatal evolution of pulmonary hypertension (PH) and cardiac dysfunction (CD). Both pathologies are important contributors to short- and long-term morbidity in these infants. The aim of the present retrospective single-center cohort study was to evaluate the need for vasoactive treatment for PH and CD in these neonates. Methodology: In-born neonates following a complicated MC twin pregnancy admitted to the department of neonatology of the University Children’s Hospital Bonn (UKB) between October 2019 and December 2023 were screened for study inclusion. Finally, 70 neonates were included in the final analysis, with 37 neonates subclassified as recipient twins (group A) and 33 neonates as donor twins (group B). Results: The overall PH incidence at day of life (DOL) 1 was 17% and decreased to 6% at DOL 7 (*p* = 0.013), with no PH findings at DOL 28. The overall incidence of CD was 56% at DOL 1 and decreased strongly until DOL 7 (10%, *p* = 0.015), with no diagnosis of CD at DOL 28. The use of dobutamine, norepinephrine, and vasopressin at DOL 1 until DOL 7 did not differ between the subgroups, whereas the dosing of milrinone was significantly higher in Group B at DOL 1 (*p* = 0.043). Inhaled nitric oxide (iNO) was used in 16% of the cohort, and a levosimendan therapy was administered in 34% of the neonates. One-third of the cohort was treated with oral beta blockers, and in 10%, an intravenous beta blockade (landiolol) was administered. The maximum levosimendan vasoactive–inotropic score (LVIS_max_) increased from DOL 1 (12.4 [3/27]) to DOL 2 (14.6 [1/68], *p* = 0.777), with a significant decrease thereafter as measured at DOL 7 (9.5 [2/30], *p* = 0.011). Conclusion: Early PH and CD are frequent diagnoses in neonates following a complicated MC twin pregnancy, and an individualized vasoactive treatment strategy is required in the management of these infants.

## 1. Introduction

Twin-to-twin transfusion syndrome (TTTS) is a well-known and severe fetal complication in monochorionic (MC) twins, occurring in up to 10–15% of MC twin pregnancies [1,2]. The recent advantages in optimizing outcomes and decreasing mortality rates in neonates with TTTS were achieved following the era of intrauterine laser interventions, namely the selective laser photocoagulation of the communicating placental vessels (SLPCV) [3]. Multiple studies have been conducted highlighting the efficacy of SLPCV since this technique was first introduced by Quintero and colleagues, and a modification of this laser technique was evaluated in a randomized controlled trial [4,5,6]. One major focus of the diagnosis of TTTS lies on the fetal monitoring of the cardiac function of the donor or recipient twin suffering from TTTS [7]. The other focus lies on the postnatal short- and long-term monitoring of the cardiac function of surviving twins from TTTS, as cardiac function can be strongly impaired in these neonates in the first days of fetal–neonatal transition and also later in life [8,9,10,11,12]. Neonates following TTTS are at risk for long-term morbidity, as incidences of neurodevelopmental impairment or chronic kidney disease are higher than in cases of uncomplicated MC twin pregnancies [13,14]. Other known complications in MC twin pregnancies are twin anemia–polycythemia sequence (TAPS) and selective intrauterine growth restriction (sIUGR). TAPS can occur either spontaneously or as a complication after SLPCV in neonates with TTTS, with residual anastomosis leading to a large intertwin gap of hemoglobin between MC twins [15]. Fetal growth restriction, such as sIUGR, is a known contributor to neonatal morbidity, as it predisposes affected infants to the development of bronchopulmonary dysplasia (BPD) and pulmonary hypertension (PH) [16].

Especially in the postnatal neonatal transition following TTTS, echocardiographic assessment of cardiac function is challenging, as the pathologic cardiac involvement differs between donor and recipient twins. Recipient twins in TTTS are likely to present a stronger cardiac impairment, with a high degree of diastolic and systolic ventricular dysfunction and hypertrophic cardiomyopathy (HCM). Nevertheless, donor twins are likely to present a systolic dysfunction, with a decreased ventricular ejection force, following a hyperdynamic state and hypovolemia prior to SLPCV, as shown by evaluation via speckle tracking and strain analysis [17]. Another complication in TTTS twins is persistent PH of the neonate (PPHN), for which the reported incidence rates are about 3–4% [18,19,20]. Data regarding the incidence of PPHN in neonates following TAPS or sIUGR are lacking. Furthermore, data regarding the postnatal drug management of PH or cardiac dysfunction (CD) in these neonates are scarce, and more data are required in this population due to the high risk of cardiopulmonary complications after birth and the frequent need for multiple drug treatments. Potential candidates for drug treatments for PH management include inhaled nitric oxide (iNO), phosphodiesterase-3 (PDE-3) inhibitors (milrinone), phosphodiesterase-5 (PDE-5) inhibitors (intravenous or oral sildenafil), and endothelin-receptor 1 (ET-1) antagonists (bosentan), as well as new candidates such as the calcium sensitizer levosimendan, along with the possibility of selective heart rate (HR) control using intravenous agents (ivabradine, landiolol). The pharmacological and clinical effects of these drugs have been evaluated in preterm and term infants and described in previous studies [21,22,23,24,25,26,27]. Nevertheless, data regarding PH treatment in complicated MC twin pregnancies are not available. The same counts for drug treatments for CD and systemic arterial hypotension, where multiple candidates (dobutamine, milrinone, levosimendan, norepinephrine, vasopressin) have already been evaluated in preterm and term infants but not in cohorts of infants following complicated MC twin pregnancies [28].

The aim of the present study was to evaluate the need for PH and vasoactive drug treatment in neonates resulting from a complicated MC twin pregnancy in a tertiary referral center and to discuss the findings in the context of the available literature.

## 2. Material and Methods

### 2.1. Study Cohort and Ethical Approval

We conducted a retrospective cohort study between October 2019 and December 2023 with data derived from the department of neonatology of the University Children’s Hospital Bonn (UKB). The UKB is a tertiary referral center for the perinatal treatment of complicated monochorionic twin pregnancies. An MC twin pregnancy was defined as complicated when any one of the following diagnoses was made prenatally: TTTS, TAPS, or sIUGR. Inclusion criteria were as follows: preterm and term infants with history of TTTS/TAPS/sIUGR treated at our department during the observational period. Exclusion criteria were as follows: major congenital heart defect [CHD] (need for cardiac surgery or catheter intervention), out-born neonates with hospital admission to our department after birth.

Data were retrospectively reviewed from the electronic patient’s chart and documentation system Integrated Care Manager (ICM, Draeger Medical Germany GmbH, Lübeck, Germany). As the ICM documentation system was implemented in 2019, the retrospective observational time-period started in October 2019. The study was conducted according to the guidelines of the Declaration of Helsinki and approved by the Institutional Review of the Medical Center of the UKB (local running number 2024-119-BO). Informed consent from legal guardians was waived due to the retrospective design of the study.

### 2.2. Epidemiological and Treatment Data

The following data were retrospectively recorded and included in the univariate analysis: gestational age at birth (GA), birth weight (BW), APGAR score (5 min, 10 min), sex, umbilical cord arterial pH value, CRIB Score (clinical risk index for babies), data on respiratory support (highest fraction of inspired oxygen [FiO_2_] and lowest FiO_2_ in the first 12 h of life; support with invasive mechanical ventilation [MV]), data on postnatal complications (bronchopulmonary dysplasia [BPD], intraventricular hemorrhage [IVH]), and data on the length of the in-hospital stay [LOS] and in-hospital mortality.

### 2.3. Echocardiographic Assessment and Vasoactive Drug Treatment

**Figure 1:**
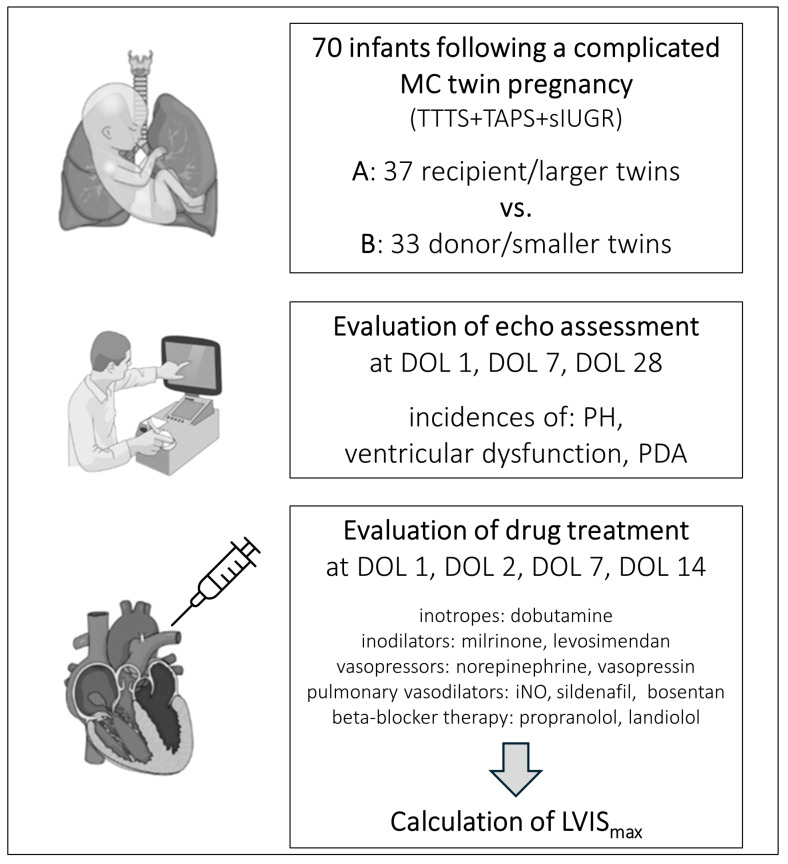
Flowchart of retrospective data collection.

A flowchart of the retrospective data collection is illustrated in Figure 1 and described in more detail below. Postnatal treatment with vasoactive drugs was due to the decision of the attending physician, according to in-hospital standards of clinical management. The echocardiographic assessment of PH or right-, left-, or bi-ventricular dysfunction (RVD, LVD, BVD) used in our department was described previously [25,27]. For the present study, the echocardiographic diagnostic reports of the attending physicians were retrospectively reviewed, and PH (moderate to severe), any cardiac dysfunction (CD), and subclassification into RVD/LVD/BVD were stated as present or not present according to the documented report/echocardiographic exam. Only neonates with moderate-to-severe PH were diagnosed as having PH, according to the following grading: (a) moderate PH: alternating PDA shunt flow (left to right/right to left), flattened interventricular septum, and tricuspid valve regurgitation II-III°; (b) severe PH: PDA shunt flow from right to left, D-shaped interventricular septum (towards the left ventricular cavity), tricuspid valve regurgitation III°. In the clinical routine, all echocardiographic assessments made by the attending physicians in our department were reviewed by a senior physician with experience in focused neonatal echocardiography, according to our in-house standard. The medical reports for day of life (DOL) 1, 7, and 28 were analyzed. As a simplification, in the following manuscript, we use the term PH as a common term for early PH (<28 DOL) and PPHN.

The choice of vasoactive drugs (classified as inotropes [dobutamine], inodilators [milrinone, levosimendan], vasopressors [norepinephrine, vasopressin], and beta blockers [propranolol, landiolol]) and drugs for PH treatment (iNO, intravenous/oral sildenafil, inhaled prostacyclin, bosentan) was made according to clinical and echocardiographic findings in the specific neonate, as described previously by our research group [25,27]. In terms of a need for beta blockade (for myocardial hypertrophy [MH] or selective HR control), neonates were treated with propranolol when oral administration was preferred and with landiolol intravenously when rapid intravenous beta blockade was necessary in the first DOL. In most neonates, landiolol, if used primarily, was later switched to oral propranolol administration. This study was not powered for the evaluation of adverse events during the drug administration; therefore, we did not report any adverse drug-related events.

For the calculation of the dosage of vasoactive drugs, a modified vasoactive–inotropic score (VIS) with consideration of the administration of levosimendan was used (LVIS), as described previously [29]. The formula for its calculation is as follows: LVIS = dobutamine dose (µg/kg/min) + 100 × epinephrine dose (µg/kg/min) + 100 × norepinephrine dose (µg/kg/min) + 10,000 × vasopressin dose (U/kg/min) + 10 × milrinone dose (µg/kg/min) + 50 × levosimendan dose (µg/kg/min). LVIS_max_, as the highest score (max) on the respective day, was retrospectively calculated for DOL 1, DOL 2, DOL 7, and DOL 14.

### 2.4. Statistical Analysis and Outcome Measures

The primary endpoint was defined as a diagnosis of PH at DOL 1. The secondary outcome measures were defined as follows: presence of PH at DOL 7/DOL 28, presence of RVD/LVD/BVD at DOL 1/DOL 7/DOL 28, LOS, duration of MV, in-hospital mortality. The cohort was subclassified when possible in recipient twins/larger twins (group A) and donor twins/smaller twins (group B). Continuous variables were described using the median and interquartile range (IQR) or the mean and minimum/maximum. Categorical variables were summarized as the absolute number (*n*) and percentage. For comparisons of continuous and non-normally distributed variables, a Wilcoxon test or Mann–Whitney U test was performed to compare continuous variables between timepoints and subgroups as appropriate. For categorical variables, Pearson’s Chi^2^ test and Fisher’s exact test were applied, as appropriate. Correlations between variables were evaluated by Spearman correlation coefficients. A *p*-value of <0.05 was considered significant. A group sample size of 37 (group A) vs. 33 (group B) was considered to be the minimum adequate sample size for this retrospective study after G-power calculation, taking into account an effect size (Cohen’s d) of 0.7, an alpha error of 0.05, a power (1-ß error) of 0.90, and an allocation ratio of subgroups of 0.89 (N^2^/N^1^: 33/37) [30]. For data analysis, SPSS version 29 (IBM Corp., Armonk, NY, USA) was used.

## 3. Results

Overall, 75 neonates (singletons or twins) following a complicated MC twin pregnancy were identified. After the exclusion of 5 neonates (3 with major CHD and 2 out-born neonates), 70 neonates were included in the final analysis. The neonates were subclassified into group A (*n* = 37) and group B (*n* = 33). The main epidemiological data are displayed in Table 1. In total, 80% of the cohort (*n* = 56) were classified as twins/singletons following TTTS, 17% (*n* = 12) following TAPS, and 3% (*n* = 2) following sIUGR. An SLPCV was performed in 39% of the cases (*n* = 27), involving 12 surviving sets of twins (*n* = 24) and 3 surviving singletons after intrauterine fetal demise (IUFD) of the co-twin. TTTS was further subclassified according to the Quintero staging into Grade 1 (34%), Grade 2 (14%), Grade 3 (17%), Grade 4 (3%), or Grade 5 (IUFD of the co-twin, 11%).

### Evaluation of Cardiac Function and Vasoactive Drug Treatment

Data on cardiac function in the cohort and subgroups are illustrated in Table 2. The overall PH incidence was 17% at DOL 1 and decreased to 6% at DOL 7. At DOL 28, no neonate had evidence of PH. No significant difference was found between recipient (larger) and donor (smaller) twins. When compared with regard to the underlying diagnosis (TTTS vs. TAPS), the PH incidence rates were as follows: DOL 1 = 16% (TTTS) vs. 17% (TAPS); DOL 7 4% (TTTS) vs. 8% (TAPS). In the twin pair following sIUGR (*n* = 2), one of the two twins was diagnosed with PH at DOL 1 and 7.

The overall incidence of CD was 56% at DOL 1 and decreased strongly until DOL 7 (10%), with no diagnosis of CD at DOL 28. Between the subgroups, there was a significant difference found at DOL 7, with a higher incidence in donor (smaller) twins (*p* = 0.045). When subclassifying CD into RVD, LVD, or BVD, no significant difference was found between the subgroups (see Table 2). At DOL 7, RVD and LVD tended to have higher values in donor/smaller twins. When again compared with regard to the underlying diagnosis (TTTS vs. TAPS), the CD incidence rates were as follows: DOL 1 = 50% (TTTS) vs. 83% (TAPS); DOL 7 5% (TTTS) vs. 25% (TAPS). In the twin pair following sIUGR (*n* = 2), one of the two twins was diagnosed with CD at DOL 1 and 7.

The evaluation of the vasoactive drug treatment and drug treatment for PH as well as MH is illustrated in Table 3. The use of vasoactive drugs (dobutamine, norepinephrine, and vasopressin) from DOL 1 until DOL 7 did not differ between the subgroups, whereas the dosing of milrinone was significantly higher in donor/smaller twins at DOL 1. At DOL 14, no vasoactive treatment due to PH or CD was administered in the overall cohort. The use of levosimendan was equally distributed between the subgroups, with a levosimendan therapy administered to one-third of all neonates. The incidence of MH and beta blockade treatment (propranolol) differed significantly between the subgroups, with higher rates in recipient/larger twins. The use of landiolol (10%) was equally distributed between the subgroups.

**Figure 2:**
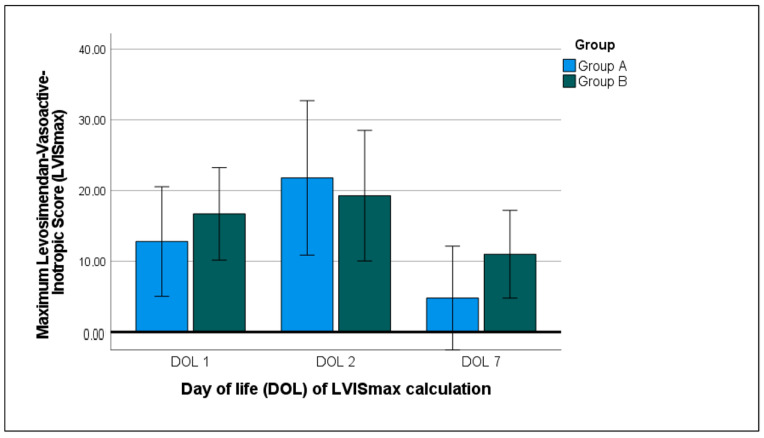
Calculation of the maximum levosimendan vasoactive–inotropic score (LVIS_max_) at DOL 1, 2, and 7 with a comparison between recipient/larger and donor/smaller twins.

The LVIS_max_ at DOL 1, 2, and 7 with the distribution between the subgroups is displayed in Figure 2. The use of vasoactive drugs increased from DOL 1 (LVIS_max_ 12.4 [3/27]) to DOL 2 (LVIS_max_ 14.6 [1/68]; *p* = 0.777), with a strong decrease in the use of vasoactive drugs to DOL 7 (LVIS_max_ 9.5 [2/30]; *p* = 0.021). No differences in the mean LVIS were found between the subgroups.

## 4. Discussion

The present retrospective cohort study gives insights into the postnatal management of PH and CD in neonates after complicated MC twin pregnancies (involving TTTS, TAPS, and sIUGR). The main findings of the present study are as follows: Both recipient/larger and donor/smaller twins were at increased risk for PH, with an overall incidence of 17% at DOL 1 and 6% at DOL 7. Furthermore, both subgroups were susceptible to CD, with every second neonate being affected at DOL 1. Many neonates following a complicated MC twin pregnancy received vasoactive and PH drug treatment, as well as drug treatment for MH. In all neonates, PH and CD resolved prior to DOL 28, and no vasoactive or PH drug treatment was administered after DOL 14, emphasizing a high potential for cardiovascular stabilization in these individuals.

### 4.1. Postnatal Findings of PH and CD in Neonates Following Complicated MC Twin Pregnancies

When critically reviewing the available literature for the evaluation of PH in neonates following a complicated MC twin pregnancy (especially TTTS), we found two studies that were conducted at the referral center for TTTS treatment in the Netherlands/Leiden [18,19]. Both studies reported an incidence of PH of about 3–4% in neonates following TTTS, which is quite a lot lower than the incidence reported in the present cohort study. In a study conducted by Gijtenbeek et al. in 2017, the authors analyzed a cohort of 598 neonates following TTTS in a case–control study with 493 neonates following an uncomplicated MC twin pregnancy (time period 2002–2016) [18]. The authors excluded neonates with TAPS or sIUGR, which is a main difference when compared to the present study. The authors revealed that early PH (1 DOL) is more prevalent in recipient twins; in particular, those born prematurely and with anemia at birth are at higher risk for experiencing PH postnatally. However, donor twins were also found to suffer from PH after birth and significantly more often when compared to the controls. These findings are in line with the findings reported in an older study from the same study center in 2007 [19]. In a database study conducted by Gijtenbeek et al., all infants with PH secondary to lung injury and MV (BPD-PH) were excluded from the retrospective database scan [18]. In the case presentation by Delsing et al., all of the four summarized neonatal cases presented PH early after birth (DOL 1–2) [19]. Our findings are in line with these two studies as the PH incidence rates were at their highest early after birth (at DOL 1). However, the PH incidence rates in the present cohort were considerably higher as compared to the abovementioned studies, both in recipient and in donor twins. The definition of PH could have biased the calculation of PH incidence, as both the abovementioned studies defined PH according to a high FiO_2_, a requirement of MV, the use of iNO, and a PDA with right-to-left shunting. Neonates with moderate PH (sub-systemic pulmonary artery pressure) will potentially be missed according to this definition. According to our PH assessment, more neonates might be identified as suffering from PH, with a higher rate of cases with moderate PH (non-severe cases). Furthermore, as neonates with IUGR are at high risk for PH after birth, this fact will have influenced the PH incidence in our cohort [31,32]. There are no consistent data on PH assessment in neonates following TAPS, but the PH incidence in these neonates needs to be considered strikingly higher than that in neonates following uncomplicated MC twin pregnancies.

In a prospective comparative trial on uncomplicated MC twin pairs (*n* = 23) and complicated MC twin pairs (15 pairs with sIUGR, 14 pairs with TTTS), the presence of postnatal CD was assessed [12]. The authors concluded that biventricular myocardial dysfunction using speckle tracking was impaired predominantly in recipient twins following TTTS without SLPCC. According to the authors, donor twins seem to have less myocardial dysfunction compared to recipient twins. Additionally, twin pairs following sIUGR pregnancies seem to have comparable myocardial function when compared to uncomplicated controls, with no myocardial dysfunction seen with strain analysis. The authors did not present the incidence of CD for the respective subgroups, but very detailed data on strain measurements and additional functional echocardiographic measurements were provided. In a prenatal fetal echocardiographic study on twins with TTTS prior to SLPCV, the authors came the same conclusion that recipient twins are more likely to suffer from ventricular dysfunction, especially diastolic filling impairment, as measured with tissue doppler imaging and pulse-waved doppler measurements [33]. Nevertheless, donor twins also showed impaired diastolic function but to a lesser extent than recipient twins. Our findings are in contrast to both studies, as according to our data, the incidence rates of CD were almost equal between both recipient and donor twins. However, there are major differences between our study and the abovementioned studies, as the study design, composition of the subgroups, and echocardiographic assessment differed strongly among the studies.

### 4.2. Postnatal Management of PH and CD in Neonates Following Complicated MC Twin Pregnancies

To date, there are no comparable data analyzing the use of drugs for PH treatment in neonates following complicated MC twin pregnancies. We could demonstrate that recipient and donor twins frequently present a need for iNO treatment due to early PH, and second- and third-line drugs such as sildenafil (i.v./orally) might be useful in selected cases with severe PH and without a response to iNO treatment. The association of levosimendan treatment with improvements in PH and CD severity in preterm and term infants was demonstrated in two previous studies from our group [26,27]. The calcium sensitizer levosimendan has the pharmacological potential to positively influence pulmonary vascular resistance by the following mechanisms: (a) calcium sensitization with a lusitropic/inotropic/vasodilating effect, (b) phosphodiesterase-3 inhibition, and (c) the vasodilation of smooth muscle cells by affecting ATP-depending potassium channels. Levosimendan is an innovative candidate drug for neonates with a combination of PH and diastolic or systolic ventricular dysfunction. Besides levosimendan, milrinone, as a PDE-3 blocker inhibitor, was found to be an effective treatment in neonates with PH when compared to sildenafil treatment [34]. Milrinone, due to its effect on smooth muscle cells and vasodilation in the pulmonary circulation, effectively reduces the PVR and affects both compromised RVs and LVs when systemic vascular resistance (SVR) is elevated.

Furthermore, a treatment option in neonates with PH and sinus tachycardia as well as MH is selective HR control using beta-blocking agents such as propranolol or landiolol. In a large cohort study, landiolol was shown to effectively and quickly induce HR control in preterm and term neonates with PH and CD [24]. Selective HR control may have manifold beneficial effects in neonates with PH and/or CD: it decreases the myocardial oxygen demand, optimizes ventricular filling in the diastolic phase, optimizes RV–LV coupling, and can improve RV relaxation impairment in terms of PH [35,36,37,38]. These effects might add a positive effect on neonates with TTTS/TAPS/sIUGR, who often present a combination of systolic and diastolic ventricular dysfunction and are prone to PH. In our opinion, affected infants should be evaluated for selective HR control in cases of persistent tachycardia.

According to our data, the use of inotropic agents in neonates following a complicated MC twin pregnancy was found to be high in the transition phase after birth, but no neonate had signs of CD (RVD/LVD/BVD) at DOL 28, which illustrates that cardiac function is restored and improvement can be expected in many infants. This is in line with previous findings in longitudinal echocardiographic studies in such infants, emphasizing that there is cardiac adaptation in the first days of life until days 5–7 [12]. Second, there are promising findings with restored CD and normal cardiac function at later stages (follow-up until 10 years of life) in surviving donors and recipients [39,40]. This underlines the impact of intrauterine SLPCV in neonates with TTTS. According to our data, at DOL 1, dobutamine was used in 41% of the cohort, milrinone in 39%, and levosimendan in 34%. Until DOL 7, 9% of the neonates remained on dobutamine and 14% on milrinone treatment. In donor twins, the use of milrinone was significantly higher at DOL 1 when compared to recipient twins, which is in contrast to the findings that recipient twins are more likely to suffer from ventricular dysfunction [12], emphasizing a higher need for inodilative treatment. Overall, treatment with inodilators (milrinone, levosimendan) should be considered in neonates following TTTS/TAPS/sIGUR, as inodilators offer the opportunity to treat both increased PVR/SVR and diastolic/systolic ventricular dysfunction [41,42]. Nevertheless, there is debate as to whether levosimendan adds additional and synergistic effects when used in combination with milrinone [43]. To date, there is no clear evidence for the preferential use of levosimendan against milrinone in the neonatal population.

In terms of MH, treatment with inotropic drugs such as dopamine or dobutamine is not recommended due to the increased myocardial oxygen demand and inotrope-induced MH. Therefore, levosimendan, with its non ß-adrenoceptor pharmaco-mechanism, is one of the preferred drugs in the treatment of CD in combination with MH (often seen in recipient twins), as levosimendan has anti-proliferative and anti-inflammatory effects and potentially leads to an improvement in MH [44]. As mentioned above, selective HR control with modern beta-blocking drugs such as landiolol or ivabradine is a treatment option in neonates with diastolic and systolic ventricular dysfunction. A possible target range for HR control which should be aimed for in neonates with sustained sinus tachycardia and CD is 150–170 bpm in preterm neonates born at <35 weeks of GA and 130–150 bpm in late-preterm and term neonates [24].

## 5. Limitations

Retrospective analysis bears the risk of over- or underestimation of statistical effects. As this study was conducted at a single center, our presented data need to be interpreted carefully when compared to other single-center data, as patient demographics and treatment protocols vary between centers. The evaluation of consensus guidelines for the standardized definition, diagnosis, and management of PH and CD in complicated MC twin pregnancies could be a future goal of multi-center studies. Furthermore, the interpretation of the non-standardized echocardiographic data is at risk for bias because echocardiographic assessment is, to some extent, operator-dependent, subjective, and based on qualitative grading (eyeballing). As the cohort consisted mainly of neonates with TTTS, the statements and conclusions will refer mainly to these neonates, as the subgroups of neonates with TAPS (*n* = 10) or sIUGR (*n* = 2) were rather small.

## 6. Conclusions

The present single-center retrospective cohort study gives insight into the postnatal drug management of PH and CD in neonates following a complicated MC twin pregnancy. These infants are at high risk for a combination of diastolic and systolic ventricular dysfunction. Furthermore, there is an increased incidence rate of PH in these neonates in the early transition phase after birth. An innovative vasoactive management strategy including inodilators, such as milrinone and levosimendan, and selective HR control may allow for a specialized drug treatment for these neonates. Standardized consensus guidelines are highly warranted for the diagnosis and treatment of PH and CD in these infants, to facilitate the interpretation of study results between referral treatment centers.

## Figures and Tables

**Table 1:** Epidemiological and treatment data.

Variables	Overall Cohort *n* = 70	Group A (*Recipient Twin/* *Larger Twin*) *n* = 37	Group B (*Donor Twin/* *Smaller Twin*) *n* = 33	*p*-Level
Gestational age, w	29 (27.4/32.5)	29 (26.5/33)	29 (27.5/32.4)	0.974
Female sex, *n* (%)	42 (60)	21 (57)	21 (64)	0.639
Birth weight, kg	1.2 (0.85/1.9)	1.35 (0.85/2.1)	1.14 (0.81/1.61)	0.194
APGAR 5 min	8 (7/9)	8 (7.5/8)	8 (7/9)	0.152
APGAR 10 min	9 (9/9)	9 (9/9)	9 (9/10)	0.479
Umbilical artery pH	7.4 (7.3/7.4)	7.34 (7.3/7.35)	7.32 (7.29/7.35)	0.481
CRIB Score	2.7 (0/17)	2.8 (14/105)	2.6 (17/85)	0.977
Highest FiO_2_ at DOL 1	0.29 (0.21/0.4)	0.3 (0.21/0.5)	0.25 (0.21/0.4)	0.375
Comorbidities and Treatment Data				
Intraventricular hemorrhage	13 (19)	10 (27)	3 (9)	0.063
Clinical sepsis, *n* (%)	24 (34)	13 (35)	11 (33)	0.99
Invasive MV, *n* (%)	24 (34)	13 (35)	11 (33)	0.99
Duration of MV, d	5 (3/7)	6 (3/9)	4 (2/5)	0.082
Oxygen supplementation, d	10 (3/76)	16 (3/88)	10 (3/43)	0.805
BPD at 36 weeks PMA, *n* (%)	7 (10)	6 (16)	1 (3)	0.110
In-hospital stay, d	54 (13/74)	55 (15/89)	42 (11/72)	0.592
IUFD of the co-twin, *n* (%)	8 (11)	5 (14)	3 (9)	0.714
In-hospital mortality, *n* (%)	5 (7)	2 (14)	3 (9)	0.661

Data are demonstrated as an absolute number and percentage or as a median and IQR (25/75). Clinical sepsis is defined as the requirement of an antibiotic therapy for at least 5 days and clinical signs of a bloodstream infection. Abbreviations: BPD: bronchopulmonary dysplasia, CRIB: clinical risk index for babies, DOL: day of life, MV: mechanical ventilation, FiO_2_: fraction of inspired oxygen, IUFD: intrauterine fetal demise, PMA: postmenstrual age.

**Table 2:** Echocardiographic assessment.

Variables	Overall Cohort *n* = 70	Group A (*Recipient Twin/Larger Twin*) *n* = 37	Group B (*Donor Twin/Smaller Twin*) *n* = 33	*p*-Level
PH, *n* (%)				
DOL 1	12 (17)	5 (14)	7 (21)	0.531
DOL 7	4 (6)	1 (3)	3 (9)	0.335
DOL 28	0			
Any Ventricular Dysfunction, *n* (%)				
DOL 1	39 (59)	19 (51)	20 (61)	0.478
DOL 7	7 (11)	1 (3)	6 (19)	0.045
DOL 28	0			
RVD, *n* (%)				
DOL 1	20 (29)	8 (22)	12 (36)	0.516
DOL 7	2 (3)	0	2 (6)	0.543
DOL 28	0			
LVD, *n* (%)				
DOL 1	30 (43)	16 (43)	13 (39)	0.238
DOL 7	5 (7)	1 (3)	4 (12)	0.342
DOL 28	0			
BVD, *n* (%)				
DOL 1	12 (17)	6 (16)	6 (18)	0.99
DOL 7	0			
DOL 28	0			
PDA, *n* (%) DOL 1				
LRS	51 (73)	27 (73)	24 (73)	0.959
XS	9 (13)	5 (14)	4 (12)	
RLS	7 (10)	3 (8)	4 (12)	
PDA, *n* (%) DOL 7				
LRS	6 (9)	1 (3)	5 (15)	0.054
XS	1 (1)	0	1 (3)	
RLS	0			
PDA, *n* (%) DOL 28				
LRS	3 (4)	1 (3)	2 (6)	0.590
XS	0			
RLS	0			

Data are demonstrated as an absolute number and percentage (%). Abbreviations: BVD: biventricular dysfunction, LRS: left-to-right shunting, LVD: left ventricular dysfunction, PH: pulmonary hypertension, PDA: patent ductus arteriosus, RLS: right-to-left shunting, RVD: right ventricular dysfunction, XS: bidirectional shunting.

**Table 3:** Drug treatment of cardiac dysfunction and pulmonary hypertension.

Variables	Overall Cohort *n* = 70	Group A (*Recipient Twin/* *Larger Twin*) *n* = 37	Group B (*Donor Twin/* *Smaller Twin*) *n* = 33	*p*-Level
Vasoactive Treatment				
Dobutamine DOL 1, µg/kg/min Dobutamine DOL 2, µg/kg/min Dobutamine DOL 7, µg/kg/min Dobutamine DOL 14, µg/kg/min	5 (5/5) 5 (3/10) 3 (3/4.3) 0	5 (4/8.5) 10 (4/11) 3 (3/3) 0	5 (5/5) 4 (3/7) 3 (3/5) 0	0.812 0.160 0.488
Milrinone DOL 1, µg/kg/min Milrinone DOL 2, µg/kg/min Milrinone DOL 7, µg/kg/min Milrinone DOL 14, µg/kg/min	0.5 (0.5/0.7) 0.7 (0.32/0.7) 0.5 (0.3/0.7) 0	0.35 (0.3/0.5) 0.5 (0.3/0.7) 0.3 (0.3/0.3)	0.5 (0.4/0.7) 0.7 (0.36/0.7) 0.5 (0.3/0.7)	0.043 0.301 0.412
Norepinephrine DOL 1, µg/kg/min Norepinephrine DOL 2, µg/kg/min Norepinephrine DOL 7, µg/kg/min Norepinephrine DOL 14, µg/kg/min	0.1 (0.1/0.1) 0.1 (0.1/0.2) 0.2 (0.03/0.2) 0	0 0.1 (0.05/0.25) 0.2 (0.2/0.2)	0.1 (0.1/0.1) 0.1 (0.1/0.3) 0.11 (0.03/0.11)	0.440 0.480
Vasopressin DOL 1, mU/kg/min Vasopressin DOL 2, mU/kg/min Vasopressin DOL 7, mU/kg/min Vasopressin DOL 14, mU/kg/min	0 0.3 (0.3/0.3) 0.6 (0.2/0.6) 0	0.3 (0.3/0.3) 0.6 (0.2/0.6)	0 0	
Levosimendan therapy, *n* (%)	24 (34)	13 (35)	11 (33)	0.99
Start of i.v. levosimendan, DOL	1 (1/2)	1 (1/2)	1 (1/2)	0.99
Treatment of Myocardial Hypertrophy				
Myocardial hypertrophy, *n* (%)	30 (43)	23 (62)	7 (21)	<0.001
LVOT obstruction, *n* (%)	14 (20)	11 (30)	3 (9)	0.039
Propranolol orally, *n* (%)	23 (33)	19 (51)	4 (12)	<0.001
Landiolol i.v., *n* (%)	7 (10)	4 (11)	3 (9)	0.99
PH-Treatment				
iNO therapy, *n* (%)	11 (16)	7 (19)	4 (12)	0.535
Prostacyclin inhaled, *n* (%)	1 (1)	0	1 (3)	0.471
Sildenafil i.v., *n* (%)	2 (3)	0	2 (6)	0.219
Sildenafil orally, *n* (%)	2 (3)	1 (3)	1 (3)	0.99
Bosentan, *n* (%)	0			

Data are presented as a median and IQR or an absolute number and percentage. A *p*-value of <0.05 was considered statistically significant. Levosimendan was administered at a dose of 0.2 µg/kg/min for a duration of 24 h. Abbreviations: DOL: day of life, iNO: inhaled nitric oxide, i.v.: intravenous, *n*: number, LVOT: left-ventricular outflow tract.

## Data Availability

The data that support the findings of this study are available on request from the corresponding author. The data are not publicly available due to privacy or ethical restrictions.
